# Training with Multiple Structurally Related Odorants Fails to Improve Generalization of Ammonium Nitrate Detection in Domesticated Dogs (*Canis familiaris*)

**DOI:** 10.3390/ani11010213

**Published:** 2021-01-16

**Authors:** David C. Dorman, Melanie L. Foster, Lucia Lazarowski

**Affiliations:** Department of Molecular and Biomedical Sciences, North Carolina State University, College of Veterinary Medicine, Raleigh, NC 27607, USA; mfoster@ils-inc.com (M.L.F.); lucia.lazarowski@auburn.edu (L.L.)

**Keywords:** canine, scent detection, improvised explosive device, categorical formation

## Abstract

**Simple Summary:**

Domestic dogs are used by military and police forces to detect improvised explosive devices (IEDs) and other explosives. A challenge with training explosive detection dogs is that the ingredients used by someone to make an IED can vary. It is therefore critical that dogs be able to detect an IED with unfamiliar ingredients. This ability can be improved if the dog’s training allows them to categorize similar odors together. Many IEDs are created using ammonium nitrate, which was the focus of our study. Based on preliminary odor training performance, we equally assigned dogs to two experimental groups. Dogs in the first group were trained with two odors related to ammonium nitrate, while dogs in the second group were trained to six related odors. We anticipated that dogs trained to six odors would be more likely to form a category. However, this was not the case since dogs in both experimental groups were unable to form a category that allowed them to identify a novel ammonium nitrate mixture. Based on our results, the use of authentic explosive materials likely remains the most cost-effective and efficient way to train explosive scent detection dogs.

**Abstract:**

A critical aspect of canine scent detection involves the animal’s ability to respond to odors based on prior odor training. In the current study, dogs (*n* = 12) were initially trained on an olfactory simple discrimination task using vanillin as the target odorant. Based on their performance on this task, dogs were assigned to experimental groups. Dogs in group 1 and 2 (*n* = 5 dogs/group; 1 dog/group were removed due to low motivation or high error rates) were trained with either two or six forms of ammonium nitrate (AN), respectively. Dogs were then assessed with a mock explosive with AN and powdered aluminum. Dogs in both groups failed to respond to the novel AN-aluminum odor. Mean success rates were 56 ± 5 and 54 ± 4% for groups 1 and 2, respectively. Overall, and individual dog performance was not statistically higher than chance indicating that dogs did not generalize from AN to a similar AN-based odorant at reliable levels desired for explosive detection dogs. These results suggest the use of authentic explosive materials, without the added complication of including category-learning methods, likely remains a cost-effective and efficient way to train explosive scent detection dogs.

## 1. Introduction

Many animal-based scent-training programs rely on the behavioral process of generalization [1]. In this context, generalization refers to an animal’s ability to categorize perceptually similar stimuli. Category formation allows an animal to respond to novel members of the category based on prior experience with stimuli that share similar physicochemical properties [1,2]. Stimulus generalization occurs as a function of perceptual similarity, with responses to novel stimuli decreasing as their similarity to the stimulus used to initially train the animal decreases, depicting a typical “generalization gradient” [3]. The ability to generalize can also be conceptualized as learning a general “rule” that applies to all members of a category, including novel instances, grouping different stimuli into categories or classes based on properties shared by the stimuli [4]. Stimuli in a category are not perceptually identical but possess common features that lead to a common response to all members of the class. Category formation allows animals to appropriately respond to novel, previously un-encountered stimuli without prior explicit training. Experimental investigation of category learning in animals involves first establishing class membership through differential reinforcement of various stimuli in a category [5]. In order to test whether a representative category has been formed, as opposed to learning to respond to individual stimuli based on item-specific features, novel exemplars representative of the category are tested. Successful category formation is demonstrated by positive transfer or generalization of responding to novel members of the category, whereas strict item-specific learning yields negative transfer results [5,6].

One application in which generalization may be of paramount importance is the detection of improvised explosive devices (IEDs), a major cause of military and civilian casualties [7,8]. The use of scent detection by domestic dogs remains one of the most effective methods for detecting these types of explosives [9]. Explosives encountered by scent-trained dogs can vary in concentration, chemical composition, milieu, and the presence of other odorants [10]; therefore, it would be advantageous for the animal to be able to detect variations of the odorant of interest that were not used in training.

Olfactory generalization of explosive compounds of military interest in dogs remains relatively unexplored but largely indicates that dogs tend to be highly specific to the odors with which they were trained [11,12,13]. Our laboratory has shown that dogs trained to detect the scent of chemically pure potassium chlorate (an explosive used in IED manufacture) poorly generalized this behavior to other potassium chlorate-based explosive mixtures that contained a novel component [14]. We also showed that dogs (*n* = 15) trained with pure ammonium nitrate (AN, NH_4_NO_3_) generalized at modest rates to other types of AN varying in source or form, such as fertilizer-grade AN (FAN) [15]. Such failures to generalize to novel mixtures containing an odorant that was previously used for training the animal are likely due to ‘configural processing’. This occurs when a new combination of odors is perceived as an entirely different entity due to the odorants chemically interacting with each other or overshadowing one another [1]. The ability of dogs to recognize a novel odorant can be expected to be lower when compared with their ability to detect a target odorant used in training. However, animal success at detecting a novel variant should occur at operationally relevant levels to ensure dogs detect relevant threats. Reports of failures by dogs to generalize to novel variants may be the result of training with a limited set of stimuli, narrowing their tendency to generalize [1]. Training with larger and more variable sets of stimuli is considered to be more effective in learning a common categorical rule rather than memorizing the specific training stimuli [16].

Our aim in the present study was to extend our earlier work investigating the ability of dogs to generalize from pure AN to other forms of AN, including FAN and calcium ammonium nitrate. We also evaluated different physical forms of AN, including pelleted (prilled) and ground materials. The objective of the present study was to investigate whether the ability of dogs to generalize learning from target odors used to train the dogs to related novel target odors can be facilitated by the manipulation of training parameters, namely the use of larger training sets consisting of either two or six related odorants. The goal of this work is to better understand the odor-detecting abilities of dogs in order to optimize their effectiveness in scent-detection of AN-based explosives.

## 2. Materials and Methods

### 2.1. Overview of the Study

The experimental protocol was reviewed and approved by the North Carolina State University (NCSU) Institutional Animal Care and Use Committee (IACUC) and the DoD US Army Medical Research and Materiel Command (USAMRMC) Animal Care and Use Review Office (ACURO). The study involved three main phases:

- Olfactory discrimination pre-training (phase I): Dogs (*n* = 12) were first trained on simple discrimination of vanillin (S+) and menthol (S−) using a previously developed 2-choice vanillin olfactory discrimination task [15] for initial odor discrimination training;
- Ammonium nitrate category training (phase II): The purpose of this phase was to train dogs with a variety of chemically related odors, including pure AN, FAN, and structurally related chemicals derived from one or more sources as positive, rewarded odors to establish a target stimulus class. Two experimental groups were used (*n* = 6 dogs/group initially; *n* = 5 dogs/group completed training):
  ◦ Dogs in group I were trained with 2 AN-related odors;
  ◦ Dogs in group II were trained using 6 AN-related odors;
  ◦ Positive odors were paired with unrelated, unrewarded odors as the negative comparison.
- Generalization testing (phase III): Once olfactory performance on the AN olfactory discrimination test reached a predetermined criterion (≥80% during a single session) based on percent correct responses to the category-inclusive training stimuli, transfer tests were conducted to test for category formation and generalization to a novel target odor related to the training stimuli (e.g., AN and aluminum training aid).

### 2.2. Animals and Their Husbandry

A cohort of 16 experimentally naïve intact, male, purpose-bred dogs (hound mixes) ranging from 13–17 months of age at the time of testing was acquired from Marshall BioResources (North Rose, NY). Dogs were born and raised at this vendor’s facility, where they were reported to have undergone a period of intensive socialization from approximately five to eight weeks of age (i.e., the critical period for canine socialization). The current study did not involve invasive or terminal procedures. At the conclusion of the study, dogs were either adopted as a pet or were transferred to other veterinary teaching or research programs at the university.

For the current study, dogs were transported to an AAALAC International-accredited facility at the NCSU College of Veterinary Medicine. Dogs were housed in an environmentally controlled, cinder block building containing 18 1.5 m × 2.4 m solid-floor pens, each equipped with a raised resting surface. Housing for the dogs is typical of research facilities and also emulates typical housing conditions for working dogs. The temperature set point was 22 ± 2 °C, and relative humidity kept between 30 and 70%. Enrichment was provided through access to a variety of hard rubber chew toys (Kong Company, Golden, CO) in the runs rotated on a weekly basis, predictable and positive daily human social interactions (e.g., a consistent schedule of feeding, on-leash walks, socialization, and training), and off-leash exercise and play with other cohort members for 1–2 h each day. Dogs were fed a balanced canine dry ration twice daily (Iams Mini Chunks, P & G Pet Care, Cincinnati, OH, USA) and provided water ad libitum. Dogs underwent a two-week period of acclimation, including daily individualized socialization with the research team and general monitoring to ensure dogs were well-adjusted and healthy before experimental activities began.

### 2.3. Behavioral Test Apparatus

The Toronto general testing apparatus (TGTA; CanCog Technologies, Toronto, ON, Canada) was used for training and testing [15,17]. This system is divided into two sections by stainless steel bars that separate where the stimuli were presented from where the animal was held. The bars were modified to create gates that allowed the dog’s head to access stimuli and obtain food rewards. Test stimuli were manually presented to the dog by the experimenter using a sliding plastic tray. The tray had three wells that held and presented the test stimuli to the dog. The stimuli (S+ and S−) were placed over the left and right wells of the tray. The chamber and equipment, including stimuli, were lightly sprayed with a disinfectant solution (Virkon^®^ S, E.I. DuPont de Nemours Co., Wilmington, DE, USA) and dried with a paper towel between subjects. The test apparatus was then thoroughly disinfected at the end of each day. Data were collected using DogCog™ software (CanCog Technologies) on a computer running a Windows 7 interface. The software recorded responses (as indicated by a keystroke from the experimenter), randomized stimulus and reward positions, and controlled trial timing.

### 2.4. Phase I: Olfactory Discrimination Pre-Training

All dogs were acclimated to the test system using previously described methods [17]. Acclimation also included reward approach training, object displacement shaping, and training on a visual discrimination task [17]. Twelve of the 16 dogs successfully completed these steps and advanced to olfactory discrimination pre-training.

Dogs were trained to discriminate the odor of vanillin (S+) from menthol (S−) in a 2-choice task using previously described methods [15]. Odorants (2 g) were held in nylon bags placed inside plastic Petri dishes with several pin-hole sized perforations on the lid. A single cleaning wipe (Kimwipes^®^, Kimberly Clark Corporation, Irving, TX, USA) placed over the nylon bag in the Petri dish reduced visual cues. A positive response occurred when a dog used its muzzle to move the plastic dish. Stimuli positions (right or left well) were counterbalanced across sessions, and stimuli did not appear in the same location for more than three consecutive trials. A trial began with the experimenter presenting the tray to the dog for 30 s to manipulate a dish. Food rewards (Pup-Peroni^®^ Original bacon-flavor treat; Del Monte Foods, San Francisco, CA, USA) were placed in the well underneath the dish containing the S+. An inaccessible food reward was attached under the S− to control for odor cues associated with the food reward. A correction procedure allowing dogs to continue responding to the S+ after committing an error was used for the first trial of each session. Afterward, an incorrect response terminated the trial. If a response did not occur within 30 s, the tray was withdrawn, a nonresponse was recorded, and the next trial began following a 30 s inter-trial interval (ITI). Daily training sessions (20 trials/day) were performed 4 to 5 days/week until dogs reached a two-stage criterion of ≥80% correct on a single session, followed by two consecutive sessions of ≥70% across the two sessions.

### 2.5. Phase II: Ammonium Nitrate Category Training

Because individual dog performance and error rates on the vanillin olfactory discrimination test varied (see Table 1), dogs were assigned to experimental groups using a matched-pair design. The assignment of dogs to the experimental groups was based on their performance (number of trials to criterion) in the vanillin olfactory discrimination test. Assignment to groups was balanced to ensure similar baseline performance between the two experimental groups. Methods in this phase were similar to those used for olfactory discrimination pre-training (2.4), except that dogs were trained using either two (group 1) or six (group 2) odorants as the S+. All S+ odorants included AN in various physical forms (e.g., powdered or prilled), source, purity, or composition (Table 2). Chemists at the US Naval Energetics Test and Evaluation Division at the Naval Surface Warfare Center analyzed the FAN sample using Fourier-transform infrared spectroscopy and X-ray fluorescence. Analysis of the FAN sample was consistent with a prilled material having a composition of >97% AN with negligible levels of either limestone (calcium carbonate) or silica (silicon dioxide). The calcium ammonium nitrate fertilizer sample was composed of 81% AN mixed with 19% calcium magnesium carbonate (CaMg(CO_3_)_2_).

Odorants comprising the non-AN (S−) category were chemically unrelated to AN, ammonium, or nitrate. Depending on the substance, odorants were either liquid or solid. Liquid odorants were dispensed by glass syringe onto nylon bags. Solid odorants (5 g) were placed inside of the nylon bags. Individual Petri dishes were assigned to a particular odorant and were not used for any other odorant.

For the 2-stimulus group, each S+ was presented an equal number of times throughout the session (10 times total), with randomized pairings of S+ and S−. For the 6-stimulus group, each S+ appeared 3–4 times each session, rotating presentations each day so that each odorant appeared equally across three consecutive sessions. The correction procedure was used during the first session of this phase, after which the correction procedure was removed, and a trial ended after an incorrect response. Once dogs met a criterion of >80% on a single session, training continued, but with the treat removed from the S− and dropped into the well after a response to the S+ rather than accessible underneath, in order to ensure dogs were not learning to discriminate between the odors of the accessible and inaccessible rewards. Criteria for advancing from this phase were the same as in pre-training.

### 2.6. Phase III: Generalization Testing

Generalization test sessions were similar to those in the previous phase (2.5), except unrewarded novel probe trials were inserted throughout the session. The probe odor consisted of 3 g of chemical grade AN (Sigma-Aldrich, St. Louis, MO, USA) and 0.25 g of flaked aluminum (Toyal American, Inc. Lockport, IL). The flaked aluminum was provided by chemists at the Indian Head Division Naval Surface Warfare Center (Indian Head, MD, USA) and was chosen due to AN and flaked aluminum commonly being used as components found in IEDs. Probe trials were conducted in which the AN-aluminum combination was presented, paired with aluminum alone as the novel comparison odor. A total of ten probe trials were presented over five sessions similar to that used during the AN discrimination training phase. However, two randomly selected trials per session were replaced with a probe trial. The novel test trials were unrewarded in order to eliminate within-session learning and were presented only twice per session in order to minimize the effects of extinction when unrewarded trials were used.

### 2.7. Data Analysis

Statistical methods similar to those used previously were used in order to facilitate qualitative comparisons with our previous AN study [14]. All data were visually inspected before analysis. For acquisition, the total number of errors and the total number of trials until criterion were calculated for comparisons of learning rates. Trials in which a nonresponse occurred were not counted as correct or incorrect and disqualified the session from counting toward meeting criterion. For generalization testing, the percentage of response to the probe target was compared between groups and to chance (50%). Where appropriate, the data were compared by tests for homogeneity of variance (Levene’s test) and an analysis of variance (ANOVA) that examined the effect of category size as a group factor on a test parameter. If Levene’s test was significant, the data were analyzed using Welch’s ANOVA. Statistical analyses were performed using SAS statistical software (JMP Pro 11.0, Cary, NC, USA). A probability value of 0.01 was used for Levene’s test, while *p* < 0.05 was used as the critical level of significance for all other statistical tests. Unless otherwise noted, the data presented represent mean (±SEM) values.

## 3. Results

### 3.1. Phase I: Olfactory Discrimination Pre-Training

Twelve of the original 16 dogs completed olfactory discrimination pre-training. Individual data are presented in Table 1. The mean (± SEM) error rate was 32.8 (±2.0%). There were no differences in error rates or the number of trials to complete the pre-training phase between dogs assigned to group 1 (2 odorants; 34.5% ± 3.1 error rate, 269.4 ± 46.9 trials to criteria) and group 2 (six odorants; 35.5% ± 1.2 error rate, 236.0 ± 53.4 trials to criteria), confirming that the matched-pair design resulted in groups that were equivalent in learning rate (*p*s > 0.651, ANOVA, *n* = 5 dogs/group).

### 3.2. Phase II: Ammonium Nitrate Category Training

Two dogs (one from each group) were dropped from the study during the AN training phase due to insufficient motivation, resulting in ten dogs completing the AN discrimination training phase. Individual data for the AN olfactory discrimination test are presented in Table 3. There were no significant differences between groups in mean (± SEM) number of trials to criteria (group 1: 494.4 ± 124.0; group 2: 677.4 ± 67.2; *p* = 0.24; Welch test) or error rate (group 1: 42.2 ± 5.3; group 2: 40.2 ± 4.4%; *p* = 0.74; Welch test).

### 3.3. Phase III: Generalization Testing

Ten dogs completed the AN-flaked aluminum probe testing. The mean (± SEM) response rates to the AN-aluminum probes in groups 1 and 2 were 56 ± 5 and 54 ± 4%, respectively. This difference in average performance between groups 1 and 2 was not statistically significant (F(1, 8)= 0.095, *p* = 0.77; ANOVA), and was not significantly different from chance (group 1: *t*(4) = 1.177, *p* = 0.305; group 2: *t*(4) = 1.00, *p* = 0.374; paired-samples *t*-test). Likewise, no individual dogs responded at above-chance levels (8/10, binomial test). Further, average response rates on baseline trials (i.e., non-probe trials) during the testing dropped below criterion performance levels (group 1: 63.77% ± 4.87; group 2: 62.99% ± 3.17) but did not differ between groups (*t*(8) = 0.14, *p* = 0.892).

## 4. Discussion

We have previously used the operant system used in our study to evaluate olfaction in dogs [15,18]. This system also has been used by others to evaluate cognitive function in dogs following aging, dietary manipulation, and pharmaceutical administration [19,20,21,22]. Laboratory-based studies of olfactory discrimination allow for a controlled analysis of olfactory learning and sensitivity in which a number of variables may be investigated including olfactory thresholds, rate of acquisition of learned response, and generalization to chemically related odors [9,23,24]. The present experiment relied on the use of a two-choice discrimination test apparatus and procedure for use with olfactory stimuli [15]. Dogs were initially trained to discriminate between a rewarded odor (vanillin) and an unrewarded odor (menthol) by manipulating scented objects with their nose in order to receive a food reward, which allows for a comparison to previous studies using this system. An alternative training approach could have used the odors of interest for initial olfactory discrimination training. Dogs in the current study required fewer trials than what we observed previously with adult Labrador retrievers. It is possible that the difference seen in the rate of acquisition of the vanillin olfactory discrimination task represents a breed difference, as shown by others evaluating canine olfaction [25,26,27]. This difference in acquisition needs to be interpreted with some caution since the dogs used in our previous study were trained on a visual discrimination task that included a reversal task and a delayed non-match to position task, and the inclusion of these tasks may have potentially slowed acquisition of the subsequent vanillin olfactory discrimination task. Both studies shared an important limitation, namely a small sample size that needs to be considered when considering our findings. Further, the current study used menthol as the S- which may have been a more distinct contrast to the vanillin compared to the ethanol used in the previous study. It is also likely that the dogs from the previous study, which were from a population of dogs selectively bred and trained for high-energy, off-leash search tasks, may be less suitable for performing tasks using a controlled and more restricted system compared to purpose-bred dogs that typically exhibit more docile temperaments [28]. However, the current study had a relatively high dropout rate of dogs that lost motivation to perform or were unable to acclimate to the testing environment, which may reflect other important differences. Interestingly, the two dogs that completed olfactory pre-training but were dropped during the AN discrimination training were the two highest performers (i.e., least trials to criteria) in the pre-training phase. Discontinuation in later testing was due to performance factors related to the apparent loss of motivation to participate in testing (i.e., excessive nonresponses) or consume treats rather than response errors, though the cause is unclear. However, this observation supports previous research suggesting a complex interaction between temperament and problem-solving ability [29]. These and other behavioral factors should be considered when selecting dogs for olfactory learning research as well as selecting breeds for scent detection roles [30,31].

Mastery of the olfactory discrimination task allowed further probing of generalization. A previous study showed that dogs trained to discriminate between 40 odors with or without accelerants demonstrated the ability of dogs to form a categorical rule [32]. Previous work in our laboratory has shown that olfactory discrimination training with a single source of AN did not produce generalization to similar, novel, chemically related variants of AN [15] prompting evaluation of multiple exemplar training strategies. Multiple exemplar training provides the subject with several examples of a category to be learned, making item-specific learning a more taxing and inefficient strategy, facilitating a shift to category formation instead [33]. To compare between limited exemplar and multiple exemplar training strategies, this experiment divided dogs into those presented with two or six training odors. As noted earlier, several trends appeared during the course of the experiment. First, there was a tendency towards an increase in the number of trials required to acquire the olfactory discrimination task for dogs learning six compared to two odors. Dogs in group 2 (six odors) required approximately 37% more trials to reach criteria when compared with dogs in group 1 (two odors). Although this difference was not statistically significant, the failure to identify a significant treatment effect was likely due to the small sample sizes used (*n* = 5 dogs/group). Others have noted a similar trend showing that learning an olfactory task involving multiple mixtures took longer for dogs to learn when compared to those trained using a single component [11,34].

When presented with probe trials, we found that training dogs with either two or six AN variants did not improve their ability to correctly alert to AN and flaked aluminum. The overall success rate for dogs in both groups was 54 to 56%, which was statistically equivalent to chance. Our results suggest that the training set of six odors was too small to facilitate category formation. Given the limited difference between the groups and the relative lack of any appreciable trend, we believe these negative results suggest that dogs learned the individual instances rather than the intended underlying category. Similar results were shown by DeGreeff et al. (2018), where increasing the number of AN variants in training (up to three) did not enhance generalization to other variants [35]. However, they also reported that the use of prilled AN in training might have increased the rate of generalization. Therefore, it may be that both the specific exemplars used to make up a training category and the number of exemplars in the category are important.

Our results highlight the significant challenge associated with category formation related to optimizing the number of similar odors found in a training set. As mentioned earlier, category formation (accelerant present or not) was established in dogs trained with 40 different odors [32]. However, studies evaluating the ability of dogs to detect prostate cancer in people using a large training set (50 prostate cancer samples and 67 control samples) failed to demonstrate category formation during double-blind tests that used novel samples; and illustrates dogs’ ability to memorize large numbers of specific samples [16]. Collectively, these studies indicate an inherent challenge in the design of studies, namely how large a training set is required to facilitate scent detection dogs to form categories. The present work suggests that six AN variants were too few to produce generalization to a novel AN-based target, which is substantially smaller than the relatively larger sets used in previous studies and therefore limits any conclusions about training with “large” stimulus sets. For practical applications to training detection dogs, future studies are needed to establish the optimal size for a training set that is sufficiently large enough to facilitate generalization while minimizing the number of unique odorants needed.

Surprisingly, multiple exemplar learning as performed under the conditions of our experiment may have been less effective than training with a single AN training odor. We previously showed [15] that overall, Labrador retrievers trained on pure AN generalized responding to an AN-flaked aluminum probe at a success rate greater than chance, though lower than operationally desirable levels. Importantly, we found that generalization varied significantly between the dogs in the previous study. This interindividual variability in generalization, as well as the moderate success to generalize after single-target training, was also reported by DeGreeff et al. [35]. Taken together, whether category training is more or less effective in training dogs to generalize to novel variants of odorants compared to single-target training is inconclusive.

Another possibility is that, rather than dogs having failed to learn the category, the novel probe odorant (AN and flaked aluminum) may have been too dissimilar from the exemplars used for training. Because the probe odorant consisted of a two-odorant combination, the addition of the odor of the aluminum may have altered dogs’ recognition of the AN in a number of ways. First, the odor of the aluminum may have masked, overshadowed, or altered the odor of the AN so that the AN was not identifiable in the compound. Alternatively, the novel combination of odorants may have been perceived as an entirely novel stimulus, leading to *‘configural processing’* of the whole rather than ‘*elemental processing*’ of the individual odors. All of these instances have been suggested as reasons for dogs’ failure to generalize to novel mixtures containing familiar odorants [1,11]. One strategy for increasing dogs’ generalization to novel mixtures was demonstrated by Hall et al. [11], in which dogs that were trained to respond to various AN-based mixtures showed greater generalization to novel AN mixtures than those trained to AN alone. Therefore, training dogs to detect a target against a constantly varying background may be a more efficient strategy than training dogs on a sufficiently large number of category exemplars [11].

Additionally, our observation that performance on baseline trials dropped below prior criterion levels achieved by the dogs suggests that performance was disrupted by the introduction of the unrewarded novel probes. Therefore, it is possible that this disruption also affected any potential generalization that may have been observed. In our previous study, average baseline performance remained high, though some individuals did demonstrate reductions in performance for some of the tested odors, These results highlight the importance of including baseline trials as a comparison in order to accurately evaluate test performance [24]. Nevertheless, the current finding was surprising, given that we attempted to mitigate performance disruption by spreading the probe trials across several days, intermixed with reinforced baseline trials. An alternative strategy would be to acclimate dogs to non-reinforced trials by introducing an intermittent reinforcement schedule during training to increase resistance to extinction [24], and to match training and test conditions as closely as possible. Another possibility is that the original training was not completely established, which could be addressed in future studies by requiring a lengthier criterion indicating the stability of performance.

## 5. Conclusions

To our knowledge, this study represents one of the few reports of evaluating categorical learning as it relates to explosive scent detection. Unfortunately, our results using a small sample size did not show a clear benefit to multiple exemplar training methods. Indeed, our study suggests that as the number of odors used for training increases, the number of trials needed to master an odor category likewise increases. Based on our results, the use of authentic explosive materials likely remains the most cost-effective and efficient way to train explosive scent detection dogs. However, it is possible that the results seen with the hounds in the current study may not be generalizable to operational explosive detection dogs due to differences in motivation, trainability, and experience [24].

## Figures and Tables

**Table 1:** Number of errors and trials required to reach criterion in the olfactory discrimination pre-training.

	Vanillin Olfactory Discrimination		
Dog Name	Number of Errors	Number of Trials	Error Rate (%)
Bud	78	220	35.5
Captain	44	140	31.4
Daniels	52	160	32.5
Guinness	152	440	34.6
Harper	113	280	40.4
Heineken	13	60	21.7
Hennessy	154	396	38.9
Jameson	49	140	35.0
Patron	131	340	38.5
Remy	45	191	23.6
Sake	88	220	40.0
Walker	17	79	21.5
Mean (± SEM)	78 ± 14	222 ± 35	32.8 ± 2.0

**Table 2:** Odorants used during the ammonium nitrate (AN) olfactory discrimination training.

Chemical Name	Amount	Group 1	Group 2
*Ammonium nitrate training set (S+)*			
Ammonium nitrate ground ^a^	5 g		x
Ammonium nitrate prilled ^a^	5 g	x	x
Calcium ammonium nitrate ground ^a^	5 g		x
Ammonium nitrate chemical grade ^b^	5 g	x	x
Calcium ammonium nitrate prilled ^a^	5 g		x
Ammonium nitrate fertilizer ^c^	5 g		x
*Distracting odors (S−)*			
Potassium chloride (KCl) ^b^	5 g	x	x
Sodium chloride (NaCl) ^b^	5 g		x
Sugar (sucrose; C_12_H_22_O_11_) ^d^	5 g	x	x
Epsom salt (mgso_4_) ^d^	5 g		x
Amyl acetate (CH_3_COO[CH_2_]_4_CH_3_) ^b^	4 µL		x
All-purpose sand ^d^	5 g		x

^a^ Obtained from (Indian Head Division Naval Surface Warfare Center, Indian Head, MD; ^b^ obtained from Sigma-Aldrich Chemical Co. (Milwaukee, WI); ^c^ Obtained from Weaver Fertilizer (Winston-Salem, NC); ^d^ obtained from local commercial suppliers.

**Table 3:** Number of errors required to reach criterion on the AN olfactory discrimination training.

Dog Name	Category Size	Number of Errors	Number of Trials	Error Rate (%)
Captain	2	186	480	38.75
Harper	2	90	280	32.14
Hennessy	2	426	680	62.65
Patron	2	67	180	37.22
Remy	2	341	852	40.02
Mean (±SEM)		222 ± 70	494 ± 124	42.2 ± 5.3
Bud	6	189	560	33.75
Daniels	6	390	927	42.07
Guinness	6	215	560	38.39
Jameson	6	282	680	41.47
Sake	6	299	660	45.30
Mean (±SEM)		275 ± 35	677 ± 67	40.2 ± 1.9

## Data Availability

The data presented in this study are contained within the article.
